# Genome-Wide Analysis of ROS Antioxidant Genes in Resurrection Species Suggest an Involvement of Distinct ROS Detoxification Systems during Desiccation

**DOI:** 10.3390/ijms20123101

**Published:** 2019-06-25

**Authors:** Saurabh Gupta, Yanni Dong, Paul P. Dijkwel, Bernd Mueller-Roeber, Tsanko S. Gechev

**Affiliations:** 1 Department Molecular Biology, Institute of Biochemistry and Biology, University of Potsdam, Karl-Liebknecht-Straße 24-25, Haus 20, 14476 Potsdam, Germany; bmr@uni-potsdam.de or mueller@mpimp-golm.mpg.de; 2 Institute of Fundamental Sciences, Massey University, Tennent Drive, Palmerston North 4474, New Zealand; y.dong@massey.ac.nz (Y.D.); p.dijkwel@massey.ac.nz (P.P.D.); 3 Max Planck Institute of Molecular Plant Physiology, Am Mühlenberg 1, 14476 Potsdam, Germany; 4 Center of Plant Systems Biology and Biotechnology (CPSBB), Ruski Blvd. 139, Plovdiv 4000, Bulgaria; 5 Department of Plant Physiology and Molecular Biology, University of Plovdiv, 24 Tsar Assen str., Plovdiv 4000, Bulgaria

**Keywords:** abiotic stress, desiccation, resurrection plants, ROS, ascorbate peroxidase, glutathione peroxidase, catalase, superoxide dismutase

## Abstract

Abiotic stress is one of the major threats to plant crop yield and productivity. When plants are exposed to stress, production of reactive oxygen species (ROS) increases, which could lead to extensive cellular damage and hence crop loss. During evolution, plants have acquired antioxidant defense systems which can not only detoxify ROS but also adjust ROS levels required for proper cell signaling. Ascorbate peroxidase (APX), glutathione peroxidase (GPX), catalase (CAT) and superoxide dismutase (SOD) are crucial enzymes involved in ROS detoxification. In this study, 40 putative *APX*, 28 *GPX*, 16 *CAT*, and 41 *SOD* genes were identified from genomes of the resurrection species *Boea hygrometrica*, *Selaginella lepidophylla*, *Xerophyta viscosa*, and *Oropetium thomaeum*, and the mesophile *Selaginella* *moellendorffii*. Phylogenetic analyses classified the APX, GPX, and SOD proteins into five clades each, and CAT proteins into three clades. Using co-expression network analysis, various regulatory modules were discovered, mainly involving glutathione, that likely work together to maintain ROS homeostasis upon desiccation stress in resurrection species. These regulatory modules also support the existence of species-specific ROS detoxification systems. The results suggest molecular pathways that regulate ROS in resurrection species and the role of *APX*, *GPX*, *CAT* and *SOD* genes in resurrection species during stress.

## 1. Introduction

Being sessile organisms, plants have to live in continuously changing environments which are often hostile for their growth and development. These environmental conditions include biotic stresses due to pathogen or herbivore attack, and abiotic stresses due to water shortage (drought), salinity, cold, heat, and others. During evolution, plants have established a myriad of metabolic and physiological mechanisms to combat such stresses. Stress-related metabolic activities often result in an excessive production of reactive oxygen species (ROS) which include chemical species like singlet oxygen (^1^O_2_), superoxide radicals (O_2_^•−^), hydrogen peroxide (H_2_O_2_), and hydroxyl radical (OH^•^), produced by an incomplete reduction of oxygen [1,2]. ROS are not only toxic products of metabolism; they also have important signaling functions to regulate stress responses and development [1,2]. Under conditions of abiotic or biotic stresses, ROS often increase to excessive levels in plant cells, leading to chemical modification of lipids, proteins and nucleic acids, and thereby to cellular dysfunction and eventually death of the plant [3]. A manifold antioxidant system evolved in plants allowing combating the cellular damages otherwise caused by ROS, and to fine-tune the low levels of ROS required for cell signaling. This antioxidant defense system includes both enzymatic and non-enzymatic components, where ascorbate peroxidase (APX), glutathione peroxidase (GPX), catalase (CAT), and superoxide dismutase (SOD) constitute the main enzymatic classes [1,2]. A general ROS scavenging network in plants is shown in Figure S1.

SODs (EC 1.15.1.1) are considered the first line of defense against oxidative damage [4] and they are present in all kingdoms of life [5]. SODs are ubiquitously present in plants and catalyze the conversion of superoxide anion radicals to H_2_O_2_ and O_2_. SODs are the only enzymes that scavenge superoxide radicals. They are present in different subcellular locations. In eukaryotes, SODs are broadly categorized into three classes based on their metal cofactors, namely, copper/zinc (Cu/Zn)-SOD, manganese (Mn)-SOD, and iron (Fe)-SOD. Cu/Zn-SODs are located in chloroplasts, the cytosol, and the extracellular space. Fe-SODs are generally found in chloroplasts while Mn-SODs are present in mitochondria and peroxisomes. Studies have reported eight SODs in *Arabidopsis thaliana* [6], rice (*Oryza sativa*; [7]) and sorghum (*Sorghum bicolor*; [8]). APX (EC 1.11.1.11) is a key factor of the ascorbate-glutathione cycle which is one of the most important H_2_O_2_ detoxification systems in plants. APXs belong to the class I heme-peroxidases involved in the reduction of H_2_O_2_ to water by utilizing ascorbate (AsA) as an electron donor. Genome-wide studies have shown that APXs are encoded by multigene families in plants and are generally divided into multiple groups on the basis of their subcellular localization [9,10]. Another set of enzymes important for antioxidant defense are GPXs (EC 1.11.1.9). GPXs belong to the non-heme containing peroxidases involved in the detoxification of H_2_O_2_ and organic hydroperoxides, using reduced glutathione (GSH) as electron donor [1,11]. Similar to APX proteins, various GPX family members with distinct subcellular localizations are known in higher plants [12,13]. CATs (EC 1.11.1.6) are a class of highly active antioxidant enzymes known to be ubiquitously present in almost all living organisms. The catalytic reaction of CATs involves the dismutation of two H_2_O_2_ molecules into H_2_O and O_2_ [14]. CATs are unique as they do not need another substrate as electron donor, in contrast to APXs and GPXs. Similar to APXs and GPXs, CAT proteins are encoded by a small number of genes. Genome wide studies have reported the presence of three *CAT* genes in *Arabidopsis*, maize (*Zea mays*) and tobacco (*Nicotiana tabacum*) [15]. APX, GPX, CAT and SOD proteins protect plants against a wide array of abiotic stresses [3,16,17]. The different types of *APX*, *GPX*, *CAT* and *SOD* genes exhibit tissue- and stress-specific expression patterns [10,11,12,18].

Among abiotic stresses, water deficit is one of the major factors affecting crop yield and productivity. In general, water stress can be described as drought, dehydration or desiccation. Drought refers to a (prolonged) period of time without water supply (e.g., due to lack of rain) which may or may not affect the plant’s physiology. Dehydration represents a more severe situation where water shortage leads to significant changes at the molecular, biochemical or physiological levels in plant cells. Desiccation is considered an extreme form of dehydration. The majority of angiosperm plants are dehydration sensitive and tend to die under extreme water deficit conditions [19]. However, a group of small vascular plants, known as resurrection species, exhibit the ability to tolerate almost complete desiccation of their vegetative tissues, while rapidly resuming normal physiological and metabolic activities after rehydration [20,21]. Resurrection plants have the potential to minimize the damage typically caused by oxidative stress commonly associated with tissue dehydration by expressing an array of ROS scavengers [22,23,24,25,26,27]. Resurrection species are considered as ideal models for studying mechanism of desiccation tolerance in plants. In the present study, we therefore focused our analysis on the identification of *APX*, *GPX*, *CAT* and *SOD* gene families in four sequenced resurrection species, namely, *Boea hygrometrica* (Bunge) R. Br. [28], *Selaginella lepidophylla* (Hook. and Grev.) Spring [29], *Xerophyta viscosa* Baker [30] and *Oropetium thomaeum* (L.f.) Trin. [31]. The desiccation sensitive species *Selaginella moellendorffii* Hieron. [32] was included in the analysis to facilitate the comparative analysis of these gene families. The role of enzymatic antioxidants in these species was inferred by prediction of their subcellular locations, and analyses of phylogenetic relationships, genome distributions, structural organization, expression profiles and co-expression patterns under desiccation stress.

## 2. Results and Discussion

### 2.1. Genome-Wide Identification of APX, GPX, CAT and SOD Genes

**Figure 1:**
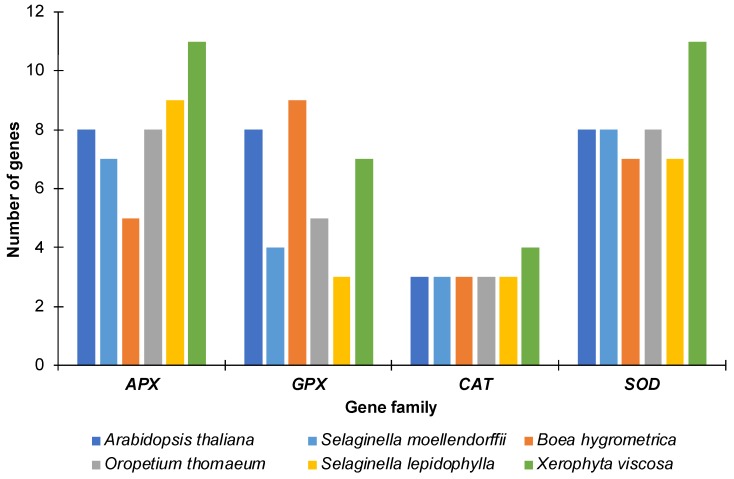
Number of genes for each family in the species analyzed. The number of genes identified for ascorbate peroxidase (APX), glutathione peroxidase (GPX), catalase (CAT) and superoxide dismutase (SOD) proteins in each species along with the number of genes found in *Arabidopsis thaliana* are represented as a bar graph.

Using similarity and Hidden Markov Model (HMM) based searches, we identified a total of 40 *APX*, 28 *GPX*, 16 *CAT* and 41 *SOD* genes in the genomes of the four resurrection and the desiccation sensitive species. These included five *APX*, nine *GPX*, three *CAT* and seven *SOD* genes in *B. hygrometrica*, eight *APX*, five *GPX*, three *CAT* and eight *SOD* genes in *O. thomaeum*, 11 *APX*, seven *GPX*, four *CAT* and 11 *SOD* genes in *X. viscosa*, nine *APX*, three *GPX*, three *CAT* and seven *SOD* genes in *S. lepidophylla*, and seven *APX*, four *GPX*, three *CAT* and eight *SOD* genes in *S. moellendorffii* (Figure 1). The lycophytes (*S. lepidophylla* and *S. moellendorffii*) contained a lower number of *GPXs* as compared to the other species including *Arabidopsis.* The highest numbers of *APX* and *SOD* genes were identified in *X. viscosa*. The larger number of antioxidant genes in higher plants likely reflects more complex signaling networks accounting for their multi-cellular nature and more elaborate morphological complexity.

The lengths of the encoded APX proteins varied from 250 to 417 amino acids (aa) in *B. hygrometrica*, 246 to 595 aa in *O. thomaeum*, 249 to 640 aa in *X. viscosa*, 249 to 506 aa in *S. lepidophylla*, and 250 to 400 aa in *S. moellendorffii* (Table S1). The average length of the identified APX proteins was 336.8 aa, which is similar to the average length of APX proteins (317.9 aa) of *Arabidopsis.* The *APX* genes encoded for polypeptides with an average molecular weight (MW) of 36.97 kDa (24.8–70.4 kDa) and an isoelectric point (pI) of 7.10 (5.16–9.03). Similar values of average MW (34.8 kDa) and pI (7.45) are found in *Arabidopsis* APXs.

In the case of the GPX family, the length of the proteins varied from 110 to 331 aa in *B. hygrometrica*, 109–245 aa in *O. thomaeum*, 103–307 aa in *X. viscosa*, 143–252 aa in *S. lepidophylla*, and 168–245 aa in *S. moellendorffii* (Table S2). The protein subcellular localization prediction analysis indicated that most GPX proteins are localized in the cytoplasm in all five species. The identified GPX proteins were broadly categorized into three main groups based on their sequence length and subcellular localization: (i) chloroplastic/mitochondrial GPXs with longer sequences (233–331 aa), (ii) extracellular/plasma membrane GPXs with medium-sized sequences (174–226 aa), (iii) cytosolic GPXs with shorter sequences (103–172 aa). The molecular weight (MW) and isoelectric point (pI) of the GPX proteins ranged from 11.4 to 37.1 kDa and from 4.8 to 10.1, respectively.

The length of the CAT proteins ranged from 243 to 488 aa in *B. hygrometrica*, 395–492 aa in *O. thomaeum*, 115–623 aa in *X. viscosa*, 492–529 aa in *S. lepidophylla*, and 484–515 aa in *S. moellendorffii* (Table S3). The average MW of the identified CATs was 50.8 kDa with an average pI of 6.82.

From a total of 41 SODs, 27 were classified as Cu/Zn-SODs and seven as Fe-SODs and Mn-SODs each (Table S4). The length of the SODs varied between 152 and 326 aa in *B. hygrometrica*, 109–242 aa in *O. thomaeum*, 117–368 aa in *X. viscosa*, 156–300 aa in *S. lepidophylla*, and 152–243 aa in *S. moellendorffii*. The *SOD* genes encoded polypeptides with an average MW of 22.0 kDa (10.9–42.1 kDa) and a pI of 6.3 (4.8–8.7).

The characteristic properties of the APX, GPX, CAT and SOD proteins including the length of the polypeptide, molecular weight (MW), theoretical isoelectric points (pI) and genomic coordinates are provided in Tables S1–S4.

### 2.2. Phylogenetic Analysis

To dissect and characterize the evolutionary relationship of the enzymatic antioxidants, phylogenetic trees were constructed using the APX, GPX, CAT and SOD proteins identified in *B. hygrometrica*, *O. thomaeum*, *X. viscosa*, *S. lepidophylla* and *S. moellendorffii* along with their orthologs from *Arabidopsis*.

For better clarity, gene names were assigned in the following manner. The first two characters describe the species, the next three the gene family, and the number that follows refers to the clade. If more than a single gene in a given species belongs to a clade, names are suffixed with a, b, c, etc., based on the order in the assembly. For example, *BhAPX1a* refers to a clade 1 *APX* gene in *B. hygrometrica* (based on the phylogenetic analysis); the suffix ‘a’ indicates the presence of more than a single *APX* gene in clade 1 of that species.

**Figure 2:**
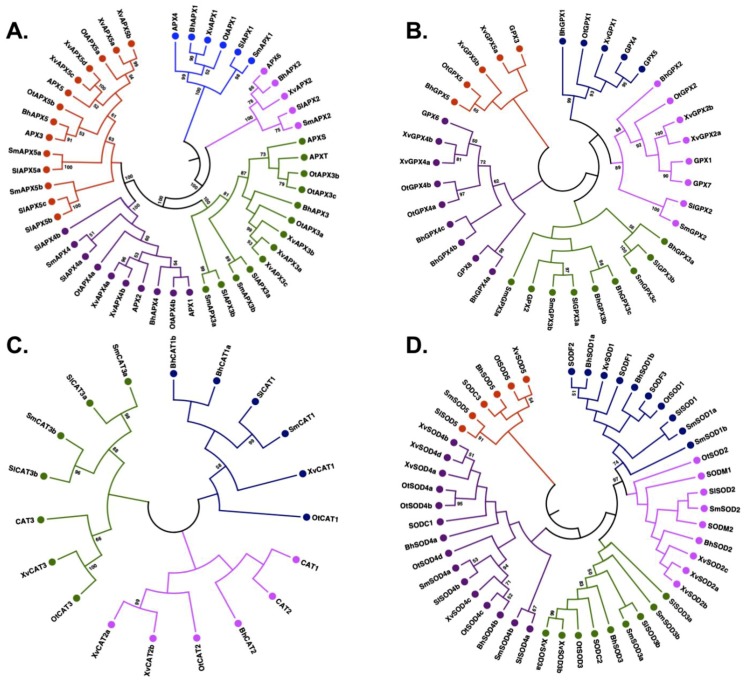
Phylogenetic analysis of APX, GPX, CAT and SOD proteins. The phylogenetic tree for each gene family was constructed by the Neighbor-joining method implemented in MEGA7 with 500 bootstraps using orthologs from *Arabidopsis thaliana*. (**A**) APX proteins were divided into five clades, (**B**) GPX proteins were divided into five clades, (**C**) CAT proteins were divide into three clades, and (**D**) SOD proteins were divided into five clades. Different clades of trees are shown in different colors: I, blue; II, pink; III, green; IV, purple, and V, red. Bootstrap values of ≥ 50 are indicated.

The phylogenetic analysis of the identified APX proteins and their orthologs from *Arabidopsis* classified them into five distinct clades (Figure 2A), in accordance with observations made in other plant species such as rice, sorghum, tomato, soybean, maize and cotton [10,33]. The members of the APX family largely clustered according to their predicted subcellular localization (Table S1). Clade I included five members with predicted chloroplastic localization, while Clade II included cytoplasmic and extracellular proteins. Clade II had four members, one from each species except *O. thomaeum*. Clade III was formed by 11 members with either cytoplasmic or chloroplastic localization. Clades IV and V encompassed members with cytoplasmic localization. Overall, APXs grouped based on their subcellular localization, in accordance with previous studies [33].

Similar to the APX family, the phylogenetic analysis classified the identified GPX members into five major clades (Figure 2B). Clade I consisted of three members with extracellular localization, one each from *B. hygrometrica*, *O. thomaeum* and *X. viscosa*, while no *GPX* gene from *S. moellendorffii* and *S. lepidophylla* was included (Table S2). Clade II consisted of six members with mitochondrial/chloroplastic localization. It was formed by one member each from the five species except *X. viscosa*, which had two *GPX* genes in this clade. The other three clades were characterized by cytoplasmic-localized GPXs (Table S2). Clade III enclosed eight members, including three from *B. hygrometrica*, three from *S. moellendorffii*, and two from *S. lepidophylla*. Clade IV was marked by seven proteins containing three members from *B. hygrometrica* and two each from *X. viscosa* and *O. thomaeum*. Two genes of *X. viscosa* and one gene each from *B. hygrometrica* and *O. thomaeum* were clustered in Clade V, together with GPX3 of *Arabidopsis*.

The phylogenetic classification of CATs divided them into three clades (Figure 2C). Clade I consisted of a total of six members including one member from all species except *B. hygrometrica* whose two CATs were part of this clade (Table S3). Interestingly, clade I did not contain any member from *Arabidopsis*, suggesting a distinct class of CAT proteins in these species. Clade II consisted of four members which included two members from *X. viscosa* and one member each from *B. hygrometrica* and *O. thomaeum*. Interestingly, none of the CATs from *S. lepidophylla* and *S. moellendorffii* were part of this clade. Clade III comprised six members formed by two members each from *S. lepidophylla* and *S. moellendorffii*, and one CAT each from *O. thomaeum* and *X. viscosa*. None of the *B. hygrometrica* CATs were clustered in clade III. The members of clade I were predicted to be mostly localized in the cytoplasm in contrast to clade II and III members which are localized in peroxisomes.

In the case of the SOD family, the phylogenetic analysis separated them into two major groups of Cu/Zn-SODs and Fe/Mn-SODs (Figure 2D). Both groups were further subdivided into a total of five clades (Table S4), in accordance with previous studies [12]. The members of clades I and II predominantly represent Fe/Mn-SODs whereas clades III, IV and V represent Cu/Zn-SODs. The maximum number of SOD proteins were clustered in clade IV (15) and the minimum in clade V (6). The SOD proteins were largely divided based on their subcellular localization. The Fe-SODs were mostly localized in chloroplasts or the cytoplasm, in contrast to Mn-SODs which were located in mitochondria or cytoplasm. The Cu-SODs were predicted to be present in the cytoplasm or targeted to chloroplasts.

Consistent with previous reports, the phylogenetic analysis showed that the proteins of these four families are mostly localized in chloroplasts, the cytoplasm, and peroxisomes, while, however, chloroplasts lack CATs [34,35]. In chloroplasts, peroxidases (instead of CATs) are responsible for scavenging H_2_O_2_ using photoreductants from thylakoids as the electron donors [36]. CAT proteins of all species studied here are predicted to be localized in peroxisomes, in accordance with the model that peroxisomes are the main organelles scavenging H_2_O_2_ [35]. The presence of different isoform families in multiple locations of the cell indicates a high level of coordination between the different cellular compartments and the different enzyme families.

### 2.3. Gene Duplication Analysis

Plant genomes have undergone several rounds of duplication events [37]. Such events have been suggested to contribute to enhanced stress tolerance of plants [38,39,40,41]. We, therefore, searched for possible duplication events in the species studied here. A total of 26 gene pairs were identified in all species (Table S5). Of the gene families analyzed, *APXs* had the highest number of paralogous pairs (13 in total). A maximum number of paralogous pairs was identified in *X. viscosa* (13), followed by *O. thomaeum* (7), *S. lepidophylla* (3) and *B. hygrometrica* (2). Interestingly, only one paralogous pair (*SmCAT3a*-*SmCAT3b*) was identified in the mesophile *S. moellendorffii*. In the case of *A. thaliana*, seven paralogous pairs were identified. This suggests that gene duplication could be an important component for stress tolerance in resurrection species. This notion is supported by the recent finding that members of the *Early Light Inducible Protein* (*ELIP*) gene family are massively expanded in number in desiccation-tolerant species [42]. ELIPs are known to protect plants against photooxidative damage under high-light conditions by chlorophyll binding and stabilizing the photosynthetic complex [43]. ELIPs are reported to be highly expressed during desiccation in resurrection species [27,44,45,46].

### 2.4. Identification of Exon-Intron Structure

We next analyzed the exon-intron structure of the *APX*, *GPX*, *CAT* and *SOD* genes. For the *APX* gene family, the number of introns ranged from 8 to 11 in *B. hygrometrica*, 6–14 in *O. thomaeum*, 6–14 in *X. viscosa*, 8–11 in *S. lepidophylla*, and 7–11 in *S. moellendorffii*. Similar structures in *APX* genes have also been reported from other plant species, such as *Sorghum bicolor* and *Gossypium hirsutum* [10,13]. The varied number of introns contributes to the variation in the length of the *APX* genes (Figure S2).

In the case of *GPXs*, the number of introns ranged from 2 to 7 in *B. hygrometrica*. Interestingly, one *GPX* gene (*BhGPX5*) was found to be intron-less. Out of five *GPX* genes identified in *O. thomaeum*, three genes had five introns and two genes had four introns. Similarly, in case of *X. viscosa* the majority of genes contained six exons and five introns. The intron number ranged from 3 to 5 and 3–6 in *S. moellendorffii* and *S. lepidophylla*, respectively. The majority of the *GPX* genes identified from the species under study consisted of six exons and five introns in concurrence with the exon-intron organization patterns observed in other plant species [47,48].

The exon-intron structure of the *CAT* genes revealed the presence of 0–6 introns in *B. hygrometrica*, 3–7 in *O. thomaeum*, 6–8 in *X. viscosa*, 6–7 in *S. lepidophylla*, and 6–9 in *S. moellendorffii*. Interestingly, *BhCAT1b* from *B. hygrometrica* was intron-less. In *Arabidopsis*, the number of introns in *CAT* genes lies between 6 and 7.

For the *SOD* gene family, the intron number varied from 4 to 7 in *B. hygrometrica*, 3–7 in *O. thomaeum*, 3–8 in *X. viscosa*, 4–9 in *S. lepidophylla*, and 3–7 in *S. moellendorffii*. The majority of the SOD proteins with chloroplastic or cytosolic localization had seven introns in their genes. A similar observation was made for *SODs* identified in cucumber [12].

Introns not only contribute to the evolution of gene structures, they may also add to the sub-functionalization of proteins encoded by the genes [49,50]. Additionally, intron sequences may contain regulatory information which may e.g., affect the gene’s expression patterns [51]. Considering the relatively higher average intron numbers and gene duplication rates in *APX* and *SOD* genes compared to *GPX* and *CAT*, we propose that *APX* and *SOD* genes have a higher probability than *GPXs* and *CATs* for evolving new specialized functions. The presence of introns also allows the plants to undergo alternative splicing and a change in splicing pattern has been confirmed as an abiotic stress response in plants for e.g., the response to drought stress in *Arabidopsis* and maize [52,53].

### 2.5. Structural Organization and Conservation Patterns

The peroxidase domain (PF00141), responsible for catalyzing oxidative reactions using H_2_O_2_ as electron acceptor, was conserved in all APXs of the selected species. In addition, two members of clade V, viz, SlAPX5b and SlAPX5c from *S. lepidophylla*, contained a tyrosine kinase domain (PF07714) towards the N-terminus. Motifs 3, 5, 7 and 11 were conserved in all APX proteins including those of *Arabidopsis* (Table S6). Motifs 1, 2 and 4 were present in all APX proteins except those of clade I, and motif 6 was present in all APX members except those of clade II. Motifs 8 and 13 were specifically found in clades IV and V. Motif 9 was found in all APXs except OtAPX4a, XvAPX3a and XvAPX5b. Motifs 10 and 17 were specific to clade III APX proteins. Motifs 12 and 16 were specific to clade I. Motif 15 was present in all members of clade V except SmAPX5b.

**Figure 3:**
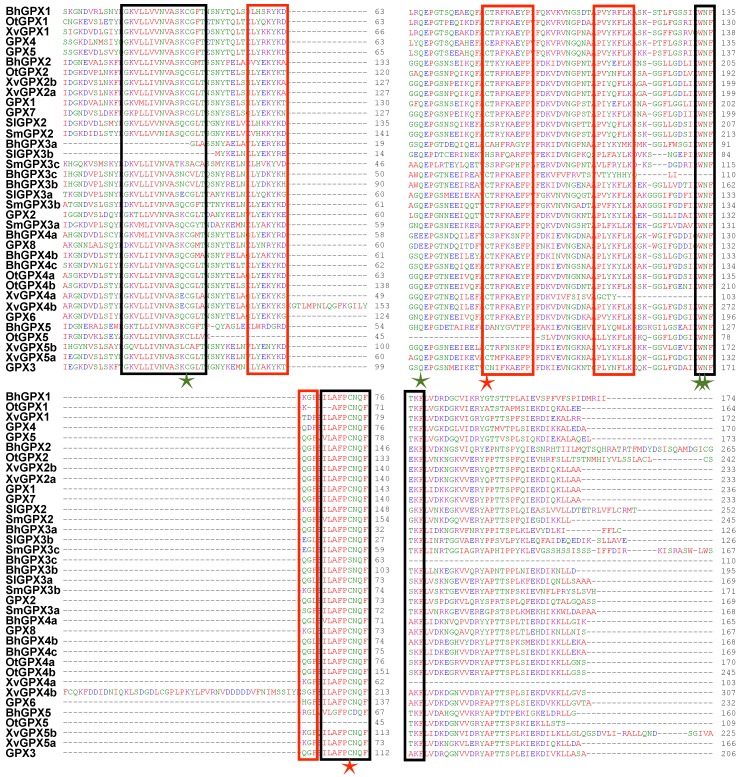
Multiple sequence alignment (MSA) of GPX proteins. MSA of the GPX members identified in *Boea hygrometrica*, *Selaginella lepidophylla*, *Xerophyta viscosa*, *Oropetium thomaeum*, and *Selaginella moellendorffii* along with the GPX proteins from *Arabidopsis thaliana* are shown. The black rectangles represent the three highly conserved GPX domains, red rectangles mark highly conserved short signatures, four green stars represent the catalytic amino acid residues and red stars represents other evolutionary conserved Cys residues.

The GPX proteins contained the approximately 100 aa long GSHPx domain (PF00255), which is necessary for GPXs to reduce lipid hydroxyperoxides to alcohols and H_2_O_2_ to water. A clade II member (*BhGPX2*) from *B. hygrometrica* encodes a GPX protein that contains the enhancer of rudimentary (ER) domain, which is highly conserved in plants and animals, towards the C-terminal end [54]. In *Drosophila*, the enhancer of rudimentary protein has been implicated in pyrimidine biosynthesis, cell cycle and to act as a positive regulator of Notch signaling [55,56]. Another protein, XvGPX4b, contained two GSHPx domains. Eight high-confidence motifs were detected in the GPXs. Motif 1 was conserved across all GPX proteins except OtGPX5, which is a partial sequence (Table S6). Motif 2 was present in all GPX proteins except three members of clade III (BhGPX3a, SlGPX3b and SmGPX3c). Similarly, motif 3 was found in all GPX proteins except BhGPX3c, XvGPX4a and OtGPX5. Motifs 1 and 3 correspond to the GSHPx domain. Motif 4 was absent in only three GPX proteins (OtGPX1, OtGPX5 and BhGPX5). Motifs 1, 2, 3 and 4 were present in all GPX proteins of *Arabidopsis*. Motif 7 was present specifically in clade II GPX proteins from *X. viscosa* (XvGPX2a and XvGPX2b). Motif 8 was present in at least one member from each species except *B. hygrometrica*. Further, the multiple sequence alignment of the identified GPX members along with GPX proteins of *Arabidopsis* revealed that the majority of the GPX proteins have three highly conserved domains; GPX signature 1, GPX signature 2, and a domain WNF(S/T)KF. Along with these conserved domains, short signature motifs were also conserved in the identified GPX proteins (Figure 3). These motifs are represented by red rectangles in the figure and include stretches such as (L/I)Y(E/D/N)(K/Q)YK(D/N)(Q/K)G(F/L) and P(V/L/I)(Y/F)(K/Q)(Y/F)LK. The GPX proteins also exhibit three cysteine (Cys) residues (marked with stars). These Cys residues are known to be highly conserved in GPX proteins from other species as well. The four highly conserved amino acid residues involved in catalytic site formation, namely Cys (C), Gln (Q), Asn (N) and Trp (W), were mostly conserved in the GPX proteins identified in the present study as well as in *Arabidopsis* GPXs (Figure 3). The conservation patterns identified through both Multiple Em for Motif Elicitation (MEME) and multiple sequence alignment are in accordance with previous studies on GPX proteins [18,48].

The CAT proteins from all species harbored a nearly 380-aa long CAT domain towards their N-terminus. The majority of the proteins also contained another conserved catalase-related immune-responsive domain (PF06628) of 61 aa length towards the C-terminus. The multiple sequence alignment showed three conserved catalytic amino acid residues (His-65, Asn-138 and Tyr-348) of CAT enzymes to be completely conserved in all CATs except BhCAT1a, BhCAT1b, OtCAT3 and XvCAT1 (Figure S3). Motifs 1 and 3 containing the catalase signature sequences, i.e., the proximal active site signature (FDRERIPERVVHARGASA) and the proximal heme-ligand signature (RIFSYADTQ), respectively, were found to be highly conserved in CATs (Figure S3; Table S6). Motif 2 was found in all CATs except two from *X. viscosa* (XvCAT1 and XvCAT2a). Motifs 4, 6, 7, 11 and 13 were conserved across clades II and III. Motif 8 was present in all clade I members except XvCAT1 and BhCAT1b. Motif 10 was absent in BhCAT1b. Motif 15 and 16 were present in clade I members of lycophytes (*S. moellendorffii* and *S. lepidophylla*) only.

Among SODs, motif 1 was conserved among members of clades III, IV and V (Table S6). This motif was also conserved in Cu-SODs of *Arabidopsis*. Motifs 2 and 6 exhibited conservation predominantly in Cu-SODs, i.e., in members of clades III, IV and V. Motifs 3, 4 and 19 were specifically found in members of clades I and II. Motif 3 represents the signature metal binding domain ’DVWEHAYY´ of Fe/Mn-SODs. This domain is conserved in Fe/Mn-SODs of other plant species as well [6,12]. Motif 7 was unique to clade I SODs, i.e., Fe-SODs. Interestingly, motif 9 was conserved in clade III and IV members of all species except SODs from *S*. *moellendorffii*. Motifs 10 and 18 were seen specifically in five members of clade IV (BhSOD4b, OtSOD4c, XvSOD4c, SlSOD4b and SmSOD4a). The clade I and II SOD members from all species except *S. lepidophylla* exhibited conservation of motif 11. Motif 17 was found specifically in two *X. viscosa* members of clade III (XvSOD3a and XvSOD3b).

The motif analysis revealed a strong conservation of specific motifs in ROS scavenging enzymes in resurrection species, although the results also suggest a substantial structural diversity among the different ROS scavenging enzyme families. The motifs are largely specific to the phylogenetic clades (Table S6). Of note, ROS species like H_2_O_2_ can diffuse between different compartments of the cell [35]; hence, this enzyme diversity likely enables plants to scavenge ROS in different locations within the cell. Such structural diversity is probably necessary for plants to adapt to different kinds of abiotic and biotic stresses. Production of ROS typically increases during abiotic stresses; therefore, a robust ROS detoxification system is required for plants to keep ROS levels in check. Such robustness is likely attained via the structural diversity of the enzymes encoded by the genes in resurrection species.

### 2.6. Prediction of Cis-Acting Elements

To better understand the transcriptional regulation of *APX*, *GPX*, *CAT* and *SOD* genes, we retrieved their 2000-bp upstream regions and analyzed them for the presence of putative *cis*-regulatory elements. A number of *cis*-acting elements were found in the promoter regions of all identified genes (Table S7). These *cis*-elements are mostly related to light, hormone and stress responses. Within the hormone-responsive category, elements related to salicylic acid, abscisic acid, methyl jasmonate, auxin and gibberellin were the most abundant. Within the stress-responsive category, *cis*-elements related to low temperature, drought, anoxia, anaerobic induction and defense responses were identified. With respect to light responses, previous studies in *Arabidopsis*, cucumber and tobacco demonstrated induction of *SOD* and *APX* genes upon high-light treatment [12,57,58,59,60]. Interestingly, TC-rich repeats involved in defense and stress responses were present in the promoters of *SOD* genes of all four resurrection species analyzed here, but absent in the mesophile *S*. *moellendorffii*. In addition, we observed several *cis*-elements having a role in developmental processes; in particular, *cis*-elements related to the control by the circadian clock, zein metabolism, meristem expression and endosperm expression were highly abundant. Common drought-responsive *cis*-elements (ABRE, DRE and MBS) are shown in Figures S4–S7. The drought-responsive *cis*-elements are highly abundant in the promoters of all four gene families in the resurrection species suggesting an involvement of the respective genes during desiccation stress.

### 2.7. miRNA Targeting of APX, GPX, CAT and SOD Genes

miRNAs are 21–22 nt long molecules known to play important roles in plant development and stress responses [61]. Several isoforms of the *miR398* and *miR8167* families were found to target *Cu*/*Zn*-*SODs* of all resurrection species analyzed here, in contrast to the mesophile *S. moellendorffii* where this pattern was not observed. In *Arabidopsis*, *miR398* targets *Cu/Zn-SODs* and plays an important role in oxidative stress tolerance [60]. A similar pattern was observed for *miR396* which targets *GPX* genes in resurrection species. In *Medicago truncatula*, *miR396* and *miR398* are down-regulated under drought stress [62]. Interestingly, *miR169*, which is up-regulated during H_2_O_2_ stress in rice [63], was found to target *GPX* and *CAT* genes in *S. moellendorffii*.

### 2.8. Expression Profiles of APX, GPX, CAT and SOD Genes in Response to Desiccation

In plants, desiccation stress leads to a myriad of changes such as the modulation of gene expression, synthesis of molecular chaperones, the accumulation of secondary metabolites, as well as the activation of proteins involved in ROS production and accumulation. To gain insight into the possible involvement of genes encoding ROS-scavenging enzymes, we therefore analyzed their expression patterns under dehydration/desiccation stress using publicly available RNA-seq datasets [28,29,30,31] (Table S8). The desiccation stress transcriptome datasets for the resurrection species varied with respect to the time points at which the samples were collected, and the magnitude of the stresses applied.

**Figure 4:**
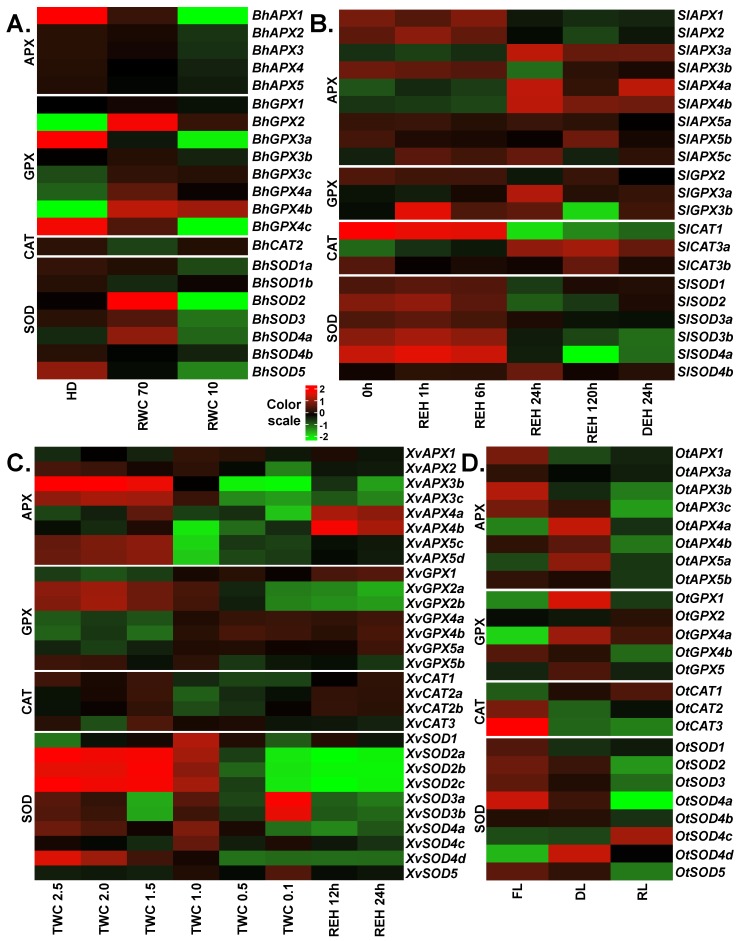
Expression profiles of *APX*, *GPX*, *CAT* and *SOD* genes upon desiccation stress in the resurrection species. The RNA-seq data from (**A**) *Boea hygrometrica*, (**B**) *Selaginella lepidophylla*, (**C**) *Xerophyta viscosa* and (**D**) *Oropetium thomaeum* were analyzed to obtain normalized expression values (TMM) for *APX*, *GPX*, *CAT* and *SOD* genes. Color scale represents the log_2_ transformed and mean centered TMM values. Genes with low or no expression values were removed. HD: fully hydrated; RWC: relative water content; 0h: three years after desiccation; REH: hours after rehydration; DEH: hours after dehydration post-rehydration; TWC: turgid water content; FL: fresh leaves; DL: desiccated leaves; RL: rehydrated leaves. RWC is calculated as (fresh weight–dry weight) × 100/(turgid weight – dry weight). TWC is the ratio of (fresh weight–dry weight) to dry weight.

In the case of *B. hygrometrica*, transcriptomic data of fully hydrated, dehydrated and desiccated samples was analyzed [28]. Upon stress, significant differences were observed for the expression of different *APX*, *GPX*, *CAT* and *SOD* genes (Figure 4A). Interestingly, all *APX* genes were down-regulated upon dehydration and desiccation stress with *BhAPX1* exhibiting the strongest down-regulation (~5.5 log_2_ fold change) upon desiccation. Among *GPXs*, three genes (*BhGPX2*, *BhGPX4a* and *BhGPX4b*) were up-regulated at both levels of stress. Upon initial stress (dehydration), these genes showed higher levels of up-regulation with log_2_ fold changes of 4.3, 1.8 and 4.3, respectively, as compared to severe stress (desiccation). An increase of *GPX* transcript levels at early stages of dehydration stress in *B. hygrometrica* has been reported [64]. Two *GPX* genes (*BhGPX3a* and *BhGPX4c*) were constitutively down-regulated upon stress. Under desiccation, these genes were down-regulated by at least four log_2_ fold change. Expression of *BhGPX5* was not detected and was hence excluded from our expression analysis. Of the three *CAT* genes in *B. hygrometrica*, only *BhCAT2* was expressed. *BhCAT2* was down-regulated upon dehydration and up-regulated upon desiccation. In the case of *SODs*, three genes (*BhSOD2*, *BhSOD3* and *BhSOD4a*) were up-regulated upon dehydration, but down-regulated at desiccation. Of note, all *SODs* exhibited a trend of down-regulation upon desiccation. Two genes, *BhSOD2* and *BhSOD5*, were down-regulated by at least 2 log_2_ fold change. *BhSOD5* was constitutively down-regulated during stress (Figure 4).

To study the role of *APX*, *GPX*, *CAT* and *SOD* genes in *O. thomaeum*, transcriptomic data from fresh, desiccated and rehydrated leaves were retrieved [31]. For *APXs*, three genes (*OtAPX4a*, *OtAPX4b* and *OtAPX5a*) were up-regulated upon desiccation. The expression of *OtAPX4a* and *OtAPX5a* was also up-regulated upon rehydration whereas *OtAPX4b* was down-regulated at rehydration. The remaining *OtAPXs* (*OtAPX1*, *OtAPX3a*, *OtAPX3b*, *OtAPX3c* and *OtAPX5b*) were down-regulated upon desiccation and rehydration. Among *GPXs*, three genes (*OtGPX1*, *OtGPX4a* and *OtGPX5*) were up-regulated upon desiccation. Similar to expression trends observed for *OtAPXs*, these three genes were up-regulated upon rehydration but to a lesser extent than during desiccation. *OtGPX4b* was down-regulated upon desiccation and rehydration. *OtCAT1* was up-regulated whereas *OtCAT2* and *OtCAT3* were down-regulated upon desiccation. In the case of *SODs*, only one *SOD*, *OtSOD4d*, was up-regulated upon desiccation. Five *SOD* genes (*OtSOD1*, *OtSOD2*, *OtSOD3*, *OtSOD4a* and *OtSOD5*) were down-regulated during desiccation and rehydration (Figure 4D).

For *S. lepidophylla*, RNA-seq data were available from leaf tissues collected after three years of desiccation, at four time points after subsequent rehydration (1, 6, and 24 h for partial recovery, and 120 h for full recovery), and a following dehydration for 24 h [29]. Among *APXs*, three genes (*SlAPX1*, *SlAPX2* and *SlAPX3b*) were down-regulated at partial and full recovery as compared to the desiccation time point, and three genes (*SlAPX3a*, *SlAPX4a* and *SlAPX4b*) were up-regulated at these time points. The expression of the remaining *APXs* did not change significantly throughout the experimental time frame (Figure 4B). Of the three *GPX* genes identified in *S. lepidophylla*, expression of *SlGPX1* was very high in the desiccated samples, as compared to other genes, and upon rehydration it was down-regulated. *SlGPX3a* was up-regulated at the recovery time-points and *SlGPX3b* was up-regulated upon rehydration and was down-regulated only in fully recovered plants. Among the genes studied, *SlCAT1* and *SlCAT3b* showed the highest expression at desiccation. *SlCAT1* was gradually down-regulated in the recovery stages and then up-regulated upon dehydration. In the case of *SODs*, all genes except *SlSOD4b* were down-regulated at the recovery time points. Similar to *GPXs*, one *SOD* (*SlSOD3a*) was very highly expressed in the desiccated samples and was down-regulated from the recovery time points onwards. Two genes (*SlSOD2* and *SlSOD4a*) were up-regulated upon dehydration as compared to the fully recovered plants. Similarly, APX, SOD and CAT enzyme activities increased upon desiccation in another resurrection species (*Selaginella bryopteris*) from the Selaginella lineage [65]. As there are currently no stress-related RNA-seq data available for *S. moellendorffii*, we could not include them in our analysis.

In the case of *X. viscosa*, we analyzed the expression profiles of *APX*, *GPX*, *CAT* and *SOD* genes using transcriptomic data from fully hydrated plants (turgid water content: TWC 2.5; TWC is the ratio of (fresh weight – dry weight) to dry weight), five stages of dehydration/desiccation stress (TWC 2.0, TWC 1.5, TWC 1.0, TWC 0.5 and TWC 0.1), and two stages of rehydration [30]. In the case of the *APX* family, three genes showed very low or no expression and hence were excluded from our expression analysis. *XvAPX1* was up-regulated upon stress and rewatering. Four genes (*XvAPX3c*, *XvAPX4a*, *XvAPX5c* and *XvAPX5d*) were slightly up-regulated at the initial stages of stress (TWC 2.0 and TWC 1.5). Among these, three genes (except *XvAPX4a*) were down-regulated upon severe stress (TWC 1.0, TWC 0.5 and TWC 0.1) and rehydration. *XvAPX3b* was down-regulated through all stages. *XvAPX4a* and XvAPX4b were generally down-regulated upon dehydration/desiccation and up-regulated upon rehydration (Figure 4C). Of *GPXs*, *XvGPX1*, *XvGPX4a*, *XvGPX4b* and *XvGPX5a* were up-regulated at severe stress and rehydration stages, while *XvGPX2a*, *XvGPX2b* and *XvGPX5b* were down-regulated. *XvGPX1* was relatively highly expressed upon rehydration as compared to the stress stages. Catalases in general were down-regulated upon stress. Clade II *CATs* (*XvCAT2a* and *XvCAT2b*) were also slightly up-regulated upon rehydration. In the case of *SODs*, *XvSOD1* was up-regulated at all stages. All members of clade II, *XvSOD2a*, *XvSOD2b* and *XvSOD2c*, were down-regulated in severe stress and during rehydration. Interestingly, both clade III *SOD* genes, *XvSOD3a* and *XvSOD3b*, were up-regulated only at the highest level of stress and down-regulated otherwise. Upon rehydration, only *XvSOD1* was up-regulated.

### 2.9. Co-Expression Network Analysis for ROS-Scavenging Genes

**Figure 5:**
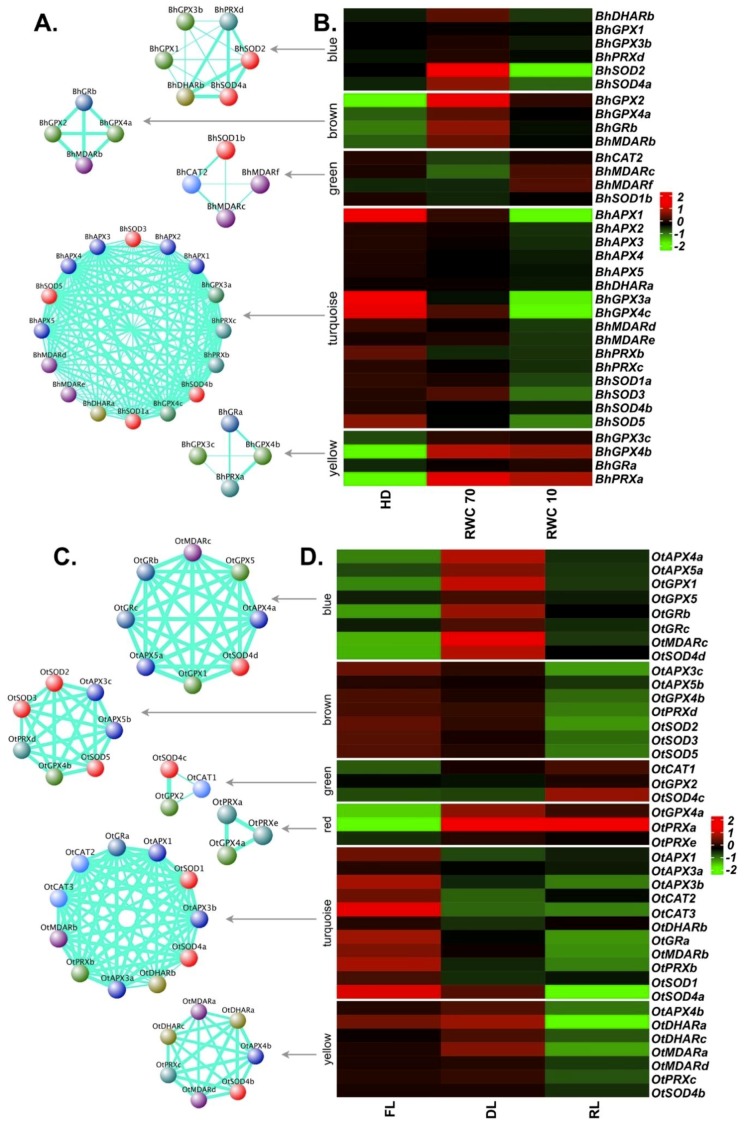
Co-expression network and expression profiles for each module of reactive oxygen species (ROS)-related genes in *B. hygrometrica* and *O. thomaeum.* (**A**,**B**) *B. hygrometrica*. (**C**,**D**) *O. thomaeum*. (**A**,**C**) Each module is represented as a co-expression network obtained from weighted gene correlation network analysis (WGCNA) analysis. Nodes correspond to a gene and are colored based on the gene family. Thickness of the edges represent the weight. Edges below adjacency threshold of 0.02 and the nodes without edges were removed. (**B**,**D**) Expression profile for all the genes in the module are plotted as a heatmap. Color scale represents the log_2_ transformed and mean centered TMM values. HD: fully hydrated; RWC: relative water content; FL: fresh leaves; DL: desiccated leaves; RL: rehydrated leaves. RWC is calculated as (fresh weight–dry weight) × 100/(turgid weight–dry weight).

APX, GPX, CAT and SOD enzymes cooperate with several other enzymes to accomplish their ROS scavenging tasks [66]. Thus, a co-expression network analysis was performed for major ROS scavengers in all resurrection species, including glutathione reductase (GR), monodehydroascorbate reductase (MDAR), peroxiredoxin (PRX) and dehydroascorbate reductase (DHAR). Based on the co-expression values, a total of five modules containing different members of ROS-related genes were identified in the case of *B. hygrometrica* (Figure 5A,B). The turquoise module was the largest with 16 genes, followed by the blue (six genes), brown, green and yellow (four genes each) modules. The turquoise module showed a pattern of down-regulation at both stages (dehydration—RWC 70, and desiccation—RWC 10) and contained all five *APX*, four *SOD*, two each of *GPX*, *MDAR*, *PRX* and one *DHAR*. The yellow module encompasses genes up-regulated at both, dehydration and desiccation stages: two *GPXs* (*BhGPX3c* and *BhGPX4b*), one *GR* (*BhGRa*) and one *PRX* (*BhPRXa*). The blue module contained genes showing up-regulation during dehydration, and down-regulation at desiccation. Two *SODs* (*BhSOD2* and *BhSOD4a*) were part of this module, along with two *GPXs* (*BhGPX1* and *BhGPX3b*), *BhDHARb* and *BhPRXd*. The brown module (*BhGPX2*, *BhGPX4a*, *BhGRb* and *BhMDARb*) also displayed a trend of up-regulation at dehydration. These two modules likely reflect the early response to drought stress. Overall, the results suggest that glutathione plays a central role in detoxifying ROS generated upon drought/desiccation stress in *B. hygrometrica*.

For *O. thomaeum*, six modules were obtained (Figure 5C,D). The largest module (turquoise: 11 genes) represented genes which were down-regulated upon desiccation and rehydration. This module contained at least one member of each family except *GPXs*. The blue module containing eight genes displayed a pattern of up-regulation at desiccation stage. It consisted of two members each from the *APX*, *GPX* and *GR*, and one member each from *MDAR* and *SOD* gene families. The red module comprised genes (*OtGPX4a*, *OtPRXa* and *OtPRXe*) with up-regulation at desiccation and rehydration stage. Altogether, this implies the major involvement of the GPX pathway in scavenging ROS in *O. thomaeum*.

**Figure 6:**
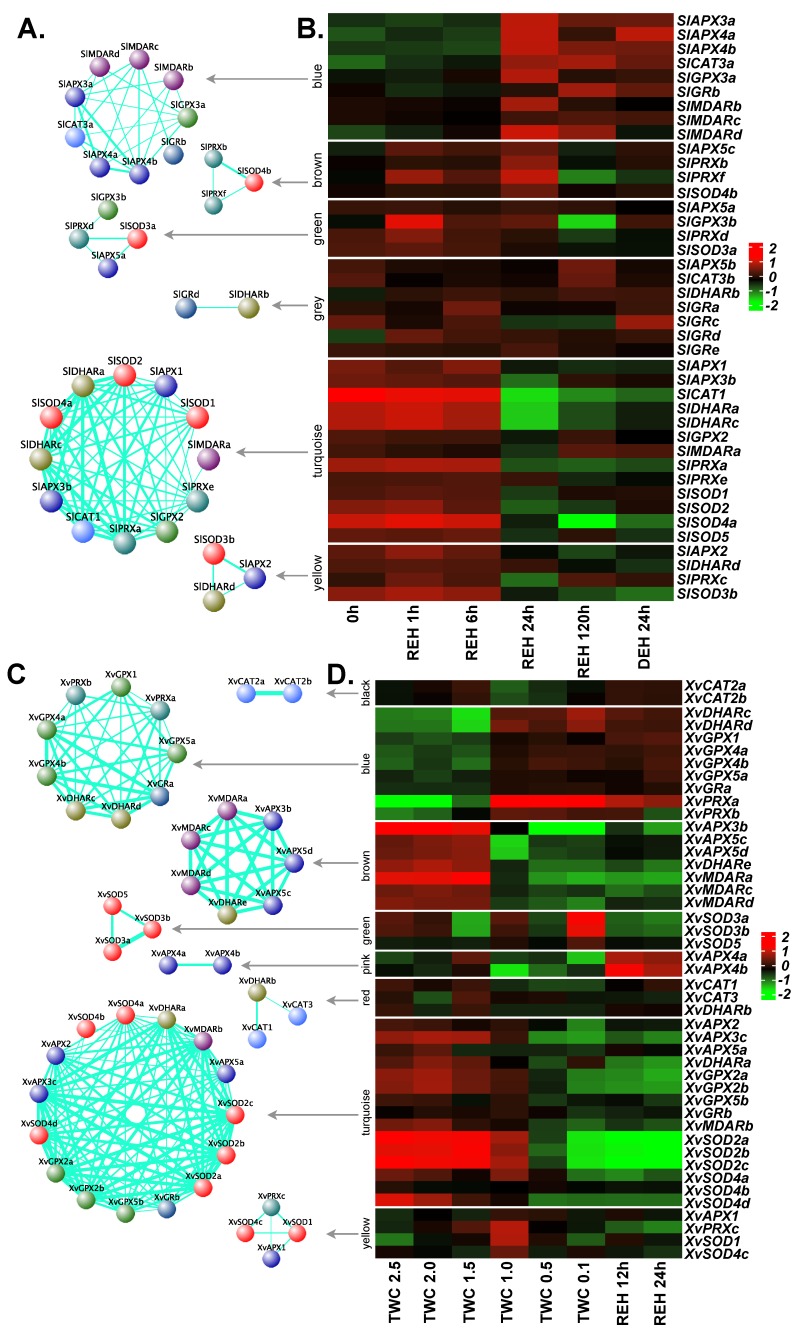
Co-expression network and expression profiles for each module of ROS-related genes in *S. lepidophylla* and *X. viscosa.* (**A**,**B**) *S. lepidophylla*. (**C**,**D**) *X. viscosa*. (**A**,**C**) Each module is represented as a co-expression network obtained from WGCNA analysis. Nodes correspond to a gene and are colored based on the gene family. Thickness of the edges represent the weight. Edges below adjacency threshold of 0.02 and the nodes without edges were removed. (**B**,**D**) Expression profile for all the genes in the module are plotted as a heatmap. Color scale represents the log_2_ transformed and mean centered TMM values. 0h: three years after desiccation; REH: hours after rehydration; DEH: hours after dehydration post-rehydration; TWC: turgid water content. TWC is the ratio of (fresh weight – dry weight) to dry weight.

In *S. lepidophylla*, the antioxidants were grouped into six modules (Figure 6A,B). The largest module, turquoise, displaying a pattern of down-regulation through recovery and full recovery stages, comprised 13 genes and contained at least one member from each family except *GRs*. In this module, *SOD*, *DHAR*, *CAT*, *APX* and *PRX* displayed a higher degree of correlation compared to *MDAR* and *GPX*. Another module (yellow) showed a similar pattern and consisted of four genes (*SlAPX2*, *SlDHARd*, *SlPRXc* and *SlSOD3b*). The blue module (nine genes: three *APXs*, three *MDARs*, one *CAT*, one *GPX*, and one *GR*) contained genes showing up-regulation at full recovery. Taken together, the co-expression analysis indicates the role of the glutathione-ascorbate cycle and PRX pathway to maintain ROS homeostasis in *S. lepidophylla*.

Eight modules were derived in *X. viscosa* with turquoise being the largest module containing 15 genes (Figure 6C,D). This module was formed by three *APX* members (*XvAPX2*, *XvAPX3c*, *XvAPX5a)*, three *GPXs* (*XvGPX2a*, *XvGPX2b*, *XvGPX5b*), and three genes representing *DHAR* (*XvDHARa*), *MDAR* (*XvMDARb*) and *GR* (*XvGRb*) families. In general, these genes were more down-regulated when the extent of the stress increased. A similar trend was evident in another module (brown) formed by three *APX*, three *MDAR* and one *DHAR* gene. The blue module displayed a trend of up-regulation during dehydration and desiccation stresses and was formed by four *GPX* (*XvGPX1*, *XvGPX4a*, *XvGPX4b*, *XvGPX5a*), two *DHAR* (*XvDHARc*, *XvDHARd*), two *PRX* (*XvPRXa*, *XvPRXb*) and one *GR* (*XvGRa*) genes. Another module (green: three genes) comprising only *SODs* (*XvSOD3a*, *XvSOD3b* and *XvSOD5*) showed a pattern of up-regulation at desiccation stage only. In contrast, the pink module containing two *APXs* (*XvAPX4a* and *XvAPX4b*) showed up-regulation during rehydration.

In summary, co-expression analysis in the resurrection species revealed that genes from the four scavenging gene families show distinct expressions patterns in response to desiccation stress, indicating there might be differences in desiccation-related ROS-scavenging mechanisms between species. The analysis also indicated potentially important roles of the ascorbate-glutathione cycle and the GPX pathway for removing excess ROS in resurrection species during desiccation stress. In many other plants, an elevated GPX activity has been observed after drought or drought-mimicking treatments [67,68,69,70]. In *A. thaliana*, one member of the *GPX* family (*ATGPX3*) plays a dual role in H_2_O_2_ homeostasis, namely as a general H_2_O_2_ scavenger and as an oxidative stress signal transducer during drought stress [71]. However, whether GPXs play a particular role for the response to drought stress in resurrection species remains to be studied. In general, the ascorbate-glutathione cycle plays a key role for H_2_O_2_ detoxification in plants responding to drought stress, which includes various free radical scavengers (such as ascorbate and glutathione) and enzymes (like SODs, ascorbate peroxidase, and glutathione reductase) [72]. This study suggests that the ascorbate-glutathione cycle also takes considerable effort to scavenge ROS-induced damage caused by drought stress in *B. hygrometrica* and *S. lepidophylla*. In summary, ascorbate and glutathione are part of a very elaborate antioxidant system, however, there appears to be a preference of glutathione to remove excess ROS in resurrection species during desiccation stress.

## 3. Materials and Methods

### 3.1. Data Acquisition

The genome sequences and annotations of all species were downloaded from respective sources (*B. hygrometrica*: NCBI [28], *O. thomaeum*: v1.0 Phytozome [31], *S. moellendorffii*: v1.0 Phytozome [32], *S. lepidophylla*: VanBuren et al., 2018 [29], *X. viscosa*: Costa et al., 2017 [30]). The quality of the genome assemblies was evaluated using BUSCO v3.0.2 (´eukaryota_odb9´ dataset; [73]) and QUAST [74]. The list of genomes analyzed in this study is provided in Table S9.

### 3.2. Identification and Characterization of APX, GPX, CAT and SOD Genes

Two different approaches were used for the genome-wide identification of *APX*, *GPX*, *CAT* and *SOD* genes: (i) the APX, GPX, CAT and SOD protein sequences of *Arabidopsis* were retrieved from the UniProtKB database (https://www.uniprot.org) and searched against the deduced protein sequences of predicted gene models using blastp (1E-20; v2.2.29+). (ii) HMM profiles of the domains corresponding to APXs (PF00141), GPXs (PF00255), SODs (PF00080; PF00081; PF02777) and CATs (PF00199) were downloaded from the Pfam database (https://pfam.xfam.org) and scanned against the deduced protein sequences of the five species analyzed here, using HMMER v3.1b2 [75] with an E-value cutoff of 1E-10. A non-redundant set of putative APX, GPX, CAT and SOD proteins identified by the above approaches were further confirmed for the presence of domains specific to each family using the SMART (http://smart.embl-heidelberg.de) and Pfam databases. The physio-chemical properties and likely subcellular localizations of the identified proteins were calculated using ProtParam (https://web.expasy.org/protparam/) and CELLO (http://cello.life.nctu.edu.tw/), respectively.

### 3.3. Phylogenetic and Gene Duplication Analysis

The protein sequences of the identified *APX*, *GPX*, *CAT* and *SOD* genes from the five species along with their homologs from *Arabidopsis* were subjected to a phylogenetic analysis. The multiple sequence alignments of the protein sequences were carried out separately using ClustalW [76] and these alignments were then used to construct a phylogenetic tree using the Neighbor-Joining method implemented in MEGA7 [77] with 500 bootstrap replicates. Paralogous gene pairs within each species were identified by sequence similarity search using blastp with query coverage ≥70%, target coverage ≥70%, percentage identity ≥70%, and an E-value ≤ 1E-20.

### 3.4. Chromosomal Distribution, Gene Structure, and Motif Analysis

The genomic coordinates of the identified *APX*, *GPX*, *SOD* and *CAT* genes were used to obtain their chromosomal locations in the respective species. Exon-intron information of the *APX*, *GPX*, *SOD* and *CAT* genes was extracted from genome annotations. The protein sequences were screened for conserved motifs using MEME from MEME Suite 5.0.2 [78] with a motif width of 10–200, a maximum number of motifs of 20, and an E-value cutoff of 0.05.

### 3.5. Promoter Analysis and miRNA Prediction Analysis

The 2000-bp sequences upstream of the translation initiation codons of *APX*, *GPX*, *CAT* and *SOD* genes were retrieved from the genome sequence based on the gene coordinates and searched for *cis*-acting elements using the PlantCARE database [79]. A matrix score of ≥5 was applied to retain high-confidence *cis*-elements. To identify microRNAs (miRNAs) targeting ROS-related transcripts, the complete set of plant miRNAs reported in miRBase [80] was searched for complementarity against transcript sequences of *APX*, *GPX*, *CAT* and *SOD* genes of all five species, using the psRNATarget server [81] with a maximum expectation value of 4.

### 3.6. Expression Profiling of APX, GPX, CAT and SOD Genes under Stress

Transcriptome datasets from desiccation stress experiments of the resurrection species were obtained from the NCBI-SRA database (https://www.ncbi.nlm.nih.gov/sra); for details of the datasets used see Table S8. All datasets used Illumina platforms for sequencing. The raw reads were processed to remove low-quality bases and trim adaptor sequences using Trimmomatic v0.35 [82]. The processed reads were aligned against a ribosomal RNA database (https://www.arb-silva.de) using SortMeRNA [83] to remove ribosomal RNA contamination. The filtered reads were pseudo-aligned against respective cDNA sequences using kallisto v0.44.0 [84]. Transcripts per million (TPM) values obtained from kallisto were further normalized to obtain trimmed means of M values (TMM; [85]). Genes with TMM values of less than 1 in more than 50% of the samples were removed. Log_2_ fold change of the genes was calculated using the edgeR package in R/Bioconductor [86]. Heatmaps were generated using ComplexHeatmap package [87]. The normalized expression values (TMM) for all genes from each dataset are provided in Table S10.

### 3.7. Co-Expression Network Analysis

To construct a co-expression network of ROS-related genes, i.e., *APX*, *GPX*, *SOD*, *CAT*, *GR*, *PRX*, *MDAR*, and *DHAR*, weighted gene correlation network analysis (WGCNA) was performed [88]. The normalized expression values (TMM) of ROS-related genes were provided as an input for construction of the co-expression network. The Pearson correlation coefficient with soft threshold power 8 was used to build the adjacency matrix for a signed network.

## 4. Conclusions

This is the first report on a genome-wide identification, characterization and comparative analysis of the major antioxidant genes, i.e., *APXs*, *GPXs*, *CATs* and *SODs*, in resurrection species. The phylogenetic analysis revealed clade- and species-specific differences within members of APX, GPX, CAT and SOD proteins based mostly on their subcellular localizations. The study demonstrates that different members of the same gene family have structural differences and exhibit different expression patterns upon stress thus highlighting their specific roles in stress responses. The differential expression patterns of these genes corroborate their involvement in desiccation stress. The co-expression analysis provides evidence for a species-specific ROS detoxification system in resurrection species with a preference for glutathione over ascorbate. This study provides a comprehensive understanding of the antioxidant gene families in resurrection plants and lays a solid foundation for deciphering the molecular mechanisms of these genes for desiccation stress acclimation. The information of ROS scavenging mechanisms in resurrection species, once confirmed, could be integrated into the existing knowledge of ROS signaling in crops to generate varieties with improved abiotic stress tolerance. Furthermore, the ROS scavenging genes from resurrection species could be transferred to high-yielding crop cultivars bearing a generally lower abiotic stress tolerance.

## Supplementary Materials

Supplementary materials can be found at https://www.mdpi.com/1422-0067/20/12/3101/s1.
