# Supplementary material for: Genome-Wide Analysis of ROS Antioxidant Genes in Resurrection Species Suggest an Involvement of Distinct ROS Detoxification Systems during Desiccation
Source: Int J Mol Sci. 2019 Jun 25;20(12):3101. doi: 10.3390/ijms20123101 (PMC6627786; doi:10.3390/ijms20123101)
Supplement: Supplementary file 1 [file ijms-20-03101-s001.zip › ijms-528876 sp for final/ijms-528876 Supplementary material figures.pdf]

Supplementary Figures

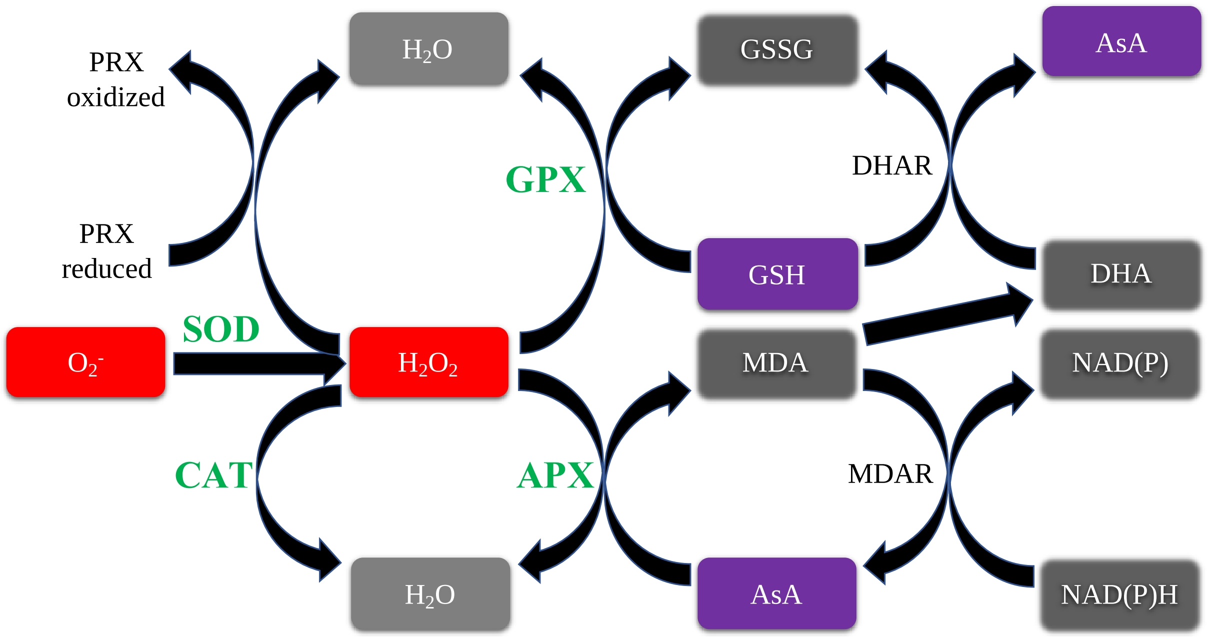

Figure S1. General ROS scavenging network in plants. Red boxes represent ROS species. The ROS scavenging enzymes studied in this report are highlighted in green. Purple boxes represent non-enzymatic antioxidants. GSSG is reduced back to GSH by the action of the enzyme glutathione reductase (GR; not shown in this figure) with NAD(P)H as electron donor. In chloroplasts, MDA can be reduced back to AsA via ferredoxin (not shown). APX: ascorbate peroxidase; GPX: glutathione peroxidase; SOD: superoxide dismutase; CAT: catalase; AsA: ascorbate; DHAR: dehydroascorbate reductase; MDAR: monodehydroascorbate reductase; MDA: monodehydroascorbate; DHA: dehydroascorbate; GSH: reduced glutathione; GSSG: oxidized glutathione; PRX: peroxiredoxin; NAD(P): nicotinamide adenine dinucleotide (phosphate); NAD(P)H: reduced form of NAD(P).

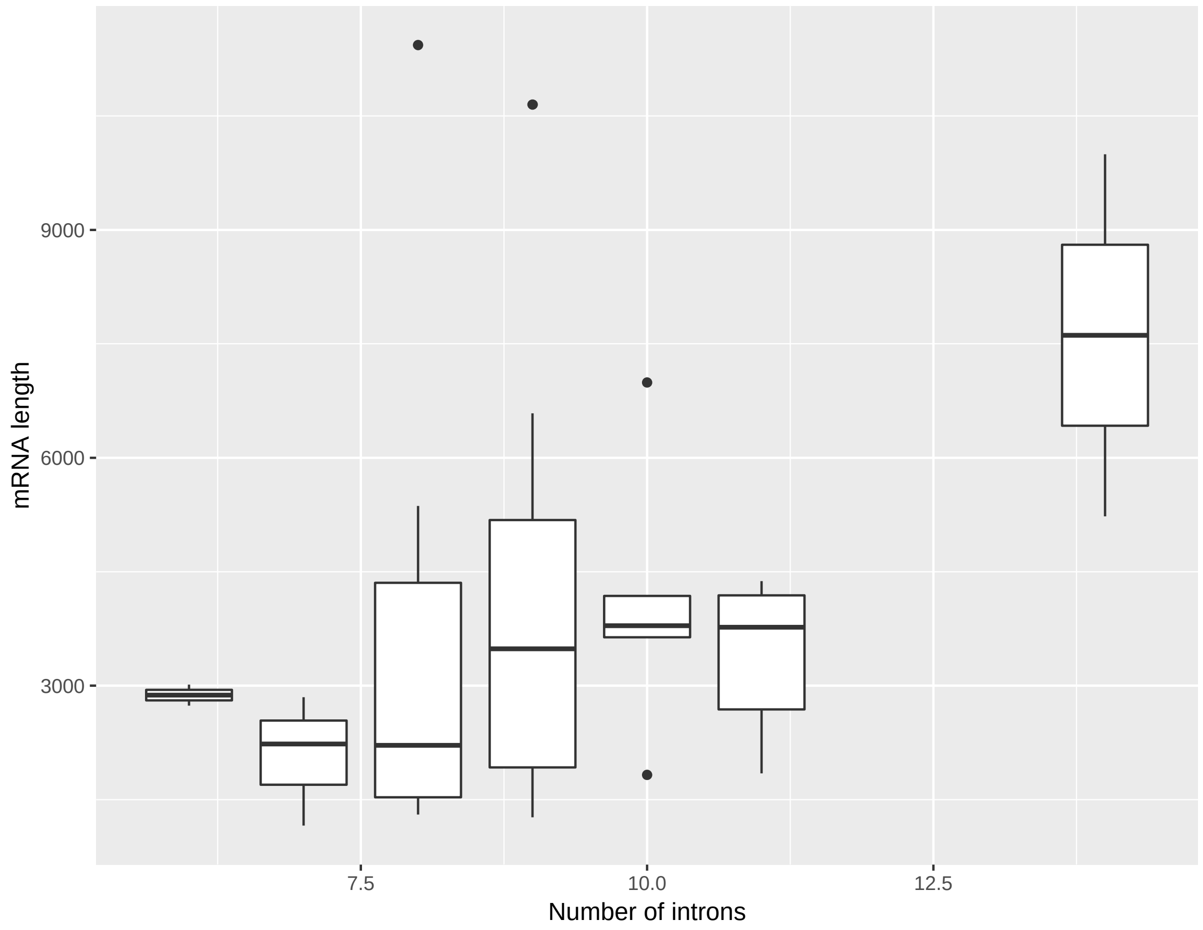

Figure S2. Length of *APX* genes vs. number of introns**.** The length of mRNAs for *APX* genes was extracted based on the gene coordinates and plotted as a boxplot against the number of introns.

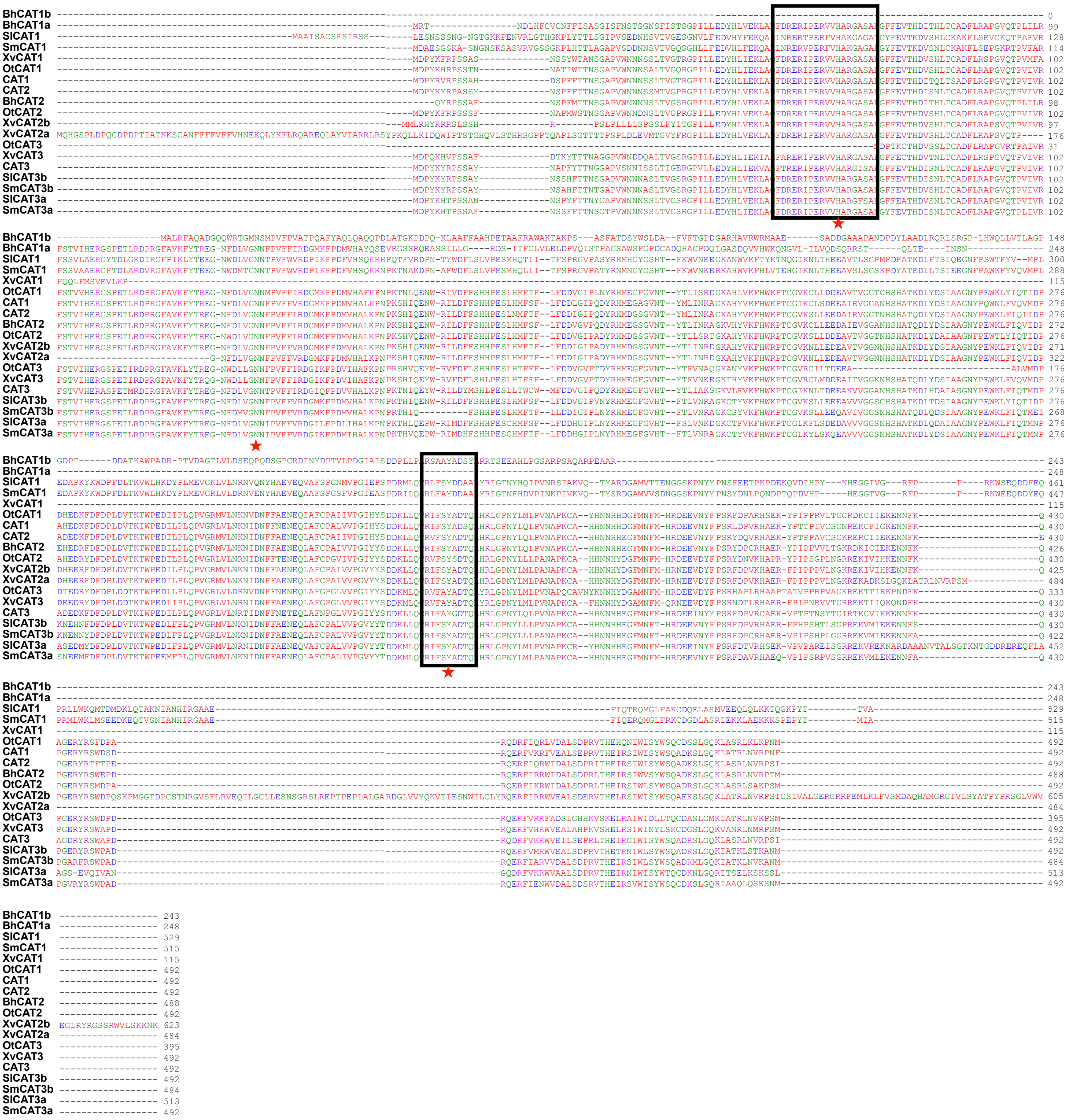

Figure S3. Multiple sequence alignment of catalase proteins. The three conserved catalytic amino acids (His, Asn and Tyr) are highlighted with red asterisks and the catalase proximal active site signature (FDRERIPERVVHAKGAGA) and proximal heme-ligand signature (RLFSYNDTH) sequences are framed.

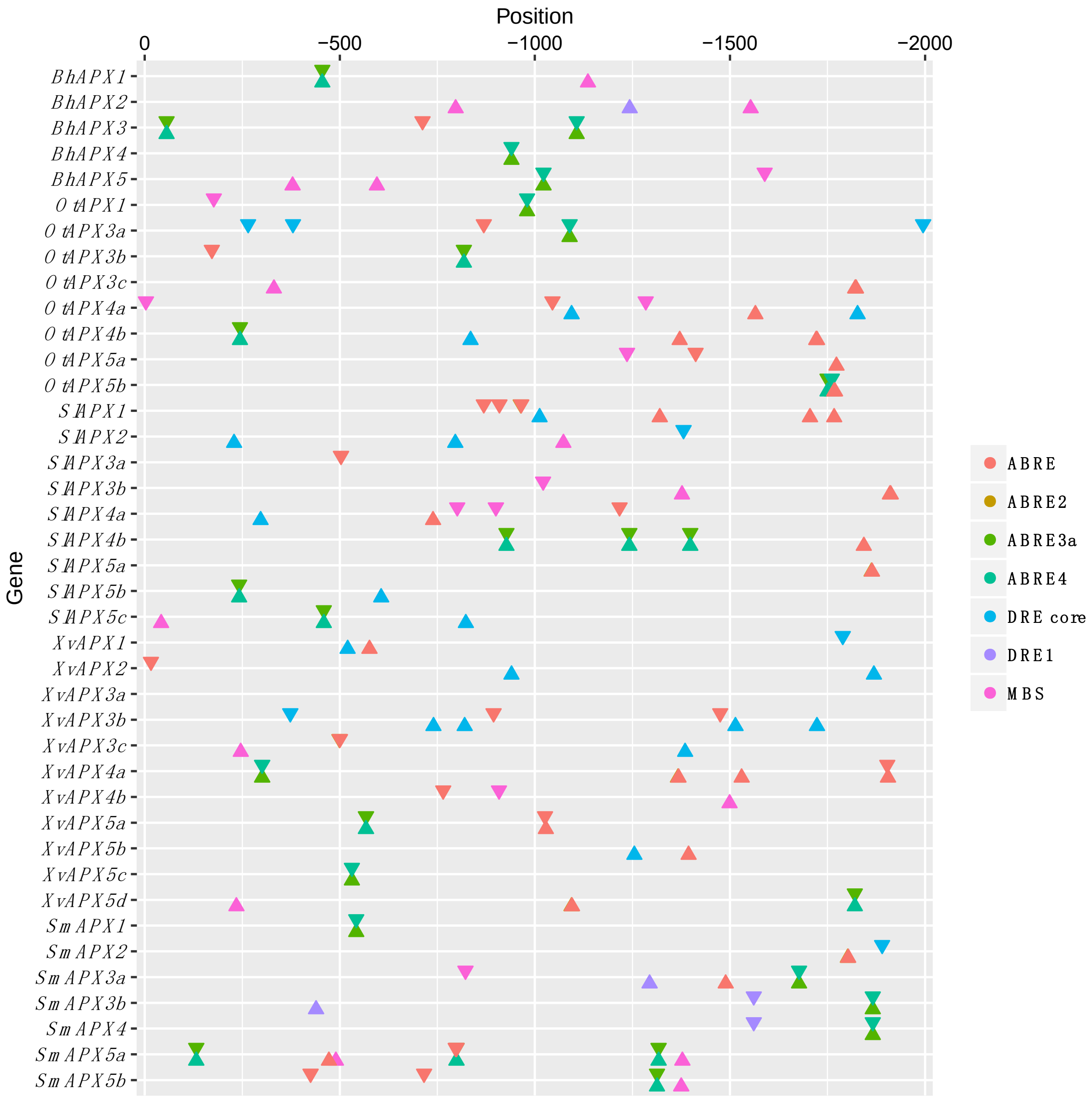

Figure S4. Distribution of drought responsive *cis*-elements in promoters of *APX* genes. Numbers on the x-axis denote distance from the transcription start site. Colors indicate the different types of *cis*-elements. Downward and upward wedges indicate that the *cis*-element is on the positive or negative strand of the promoter, respectively.

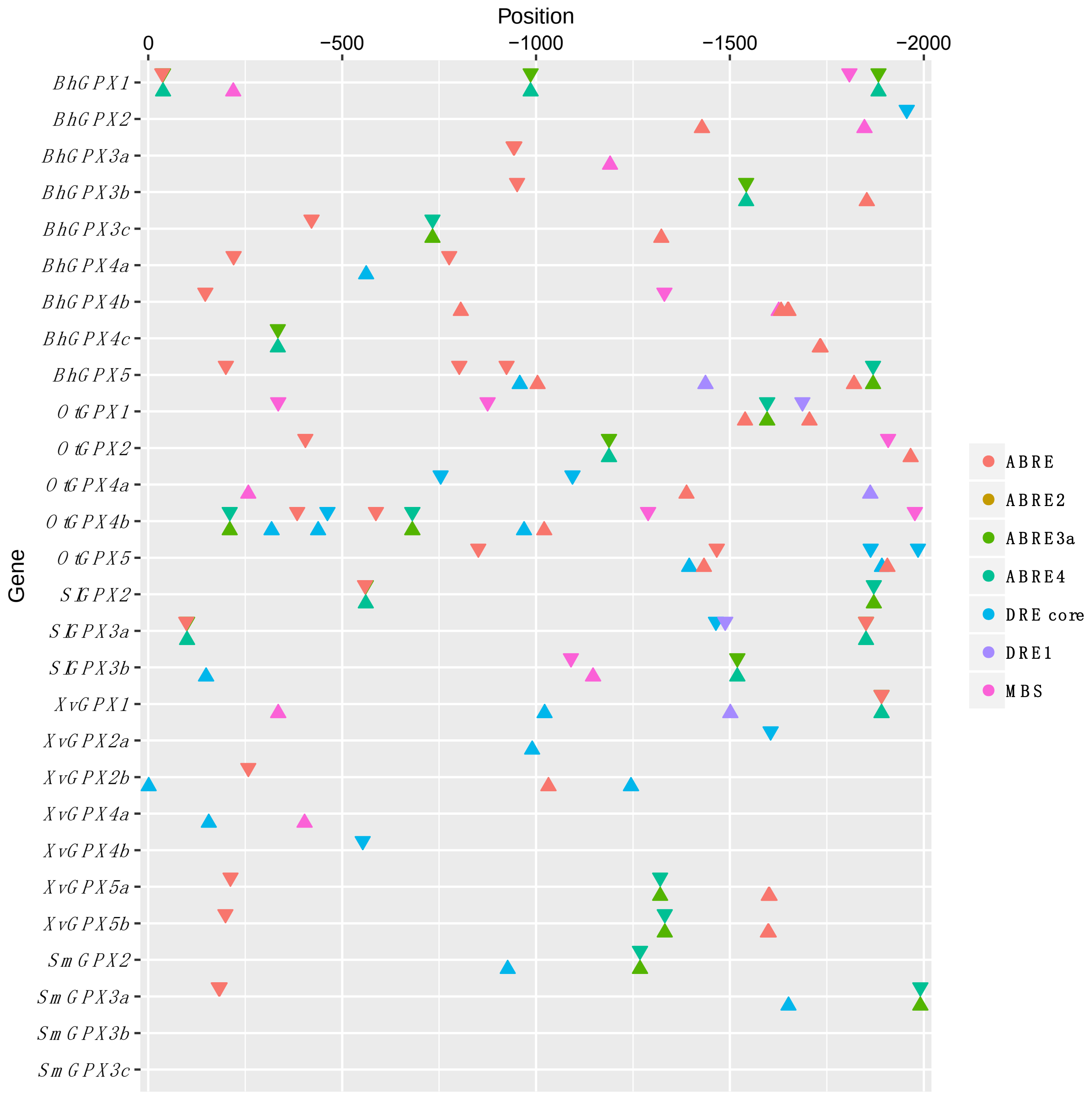

Figure S5. Distribution of drought responsive *cis*-elements in promoters of *GPX* genes. Numbers on the x-axis denote distance from the transcription start site. Colors indicate the different types of *cis*-elements. Downward and upward wedges indicate that the *cis*-element is on the positive or negative strand of the promoter, respectively.

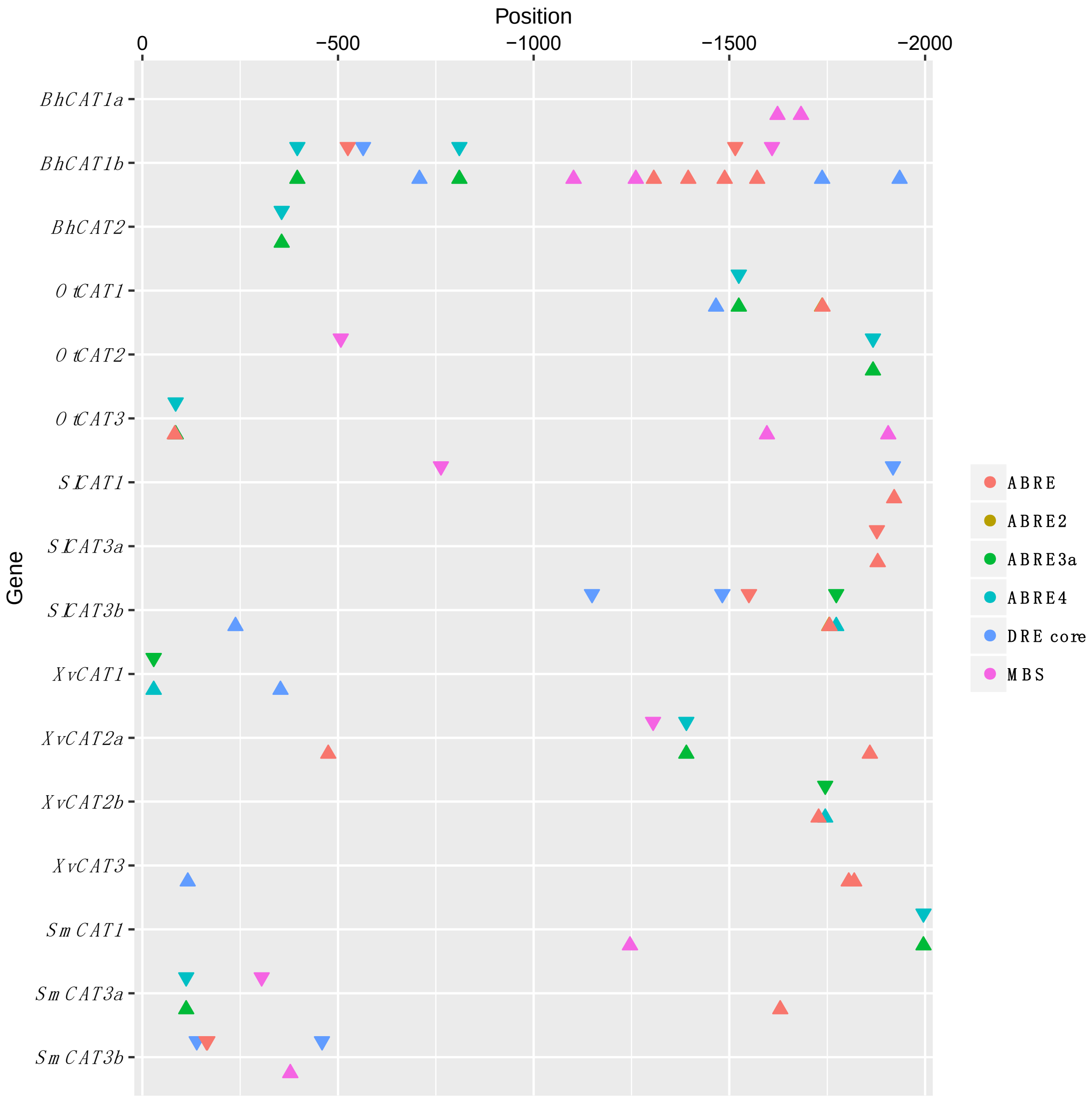

Figure S6. Distribution of drought responsive *cis*-elements in promoters of *CAT* genes. Numbers on the x-axis denote distance from the transcription start site. Colors indicate the different types of *cis*-elements. Downward and upward wedges indicate that the *cis*-element is on the positive or negative strand of the promoter, respectively.

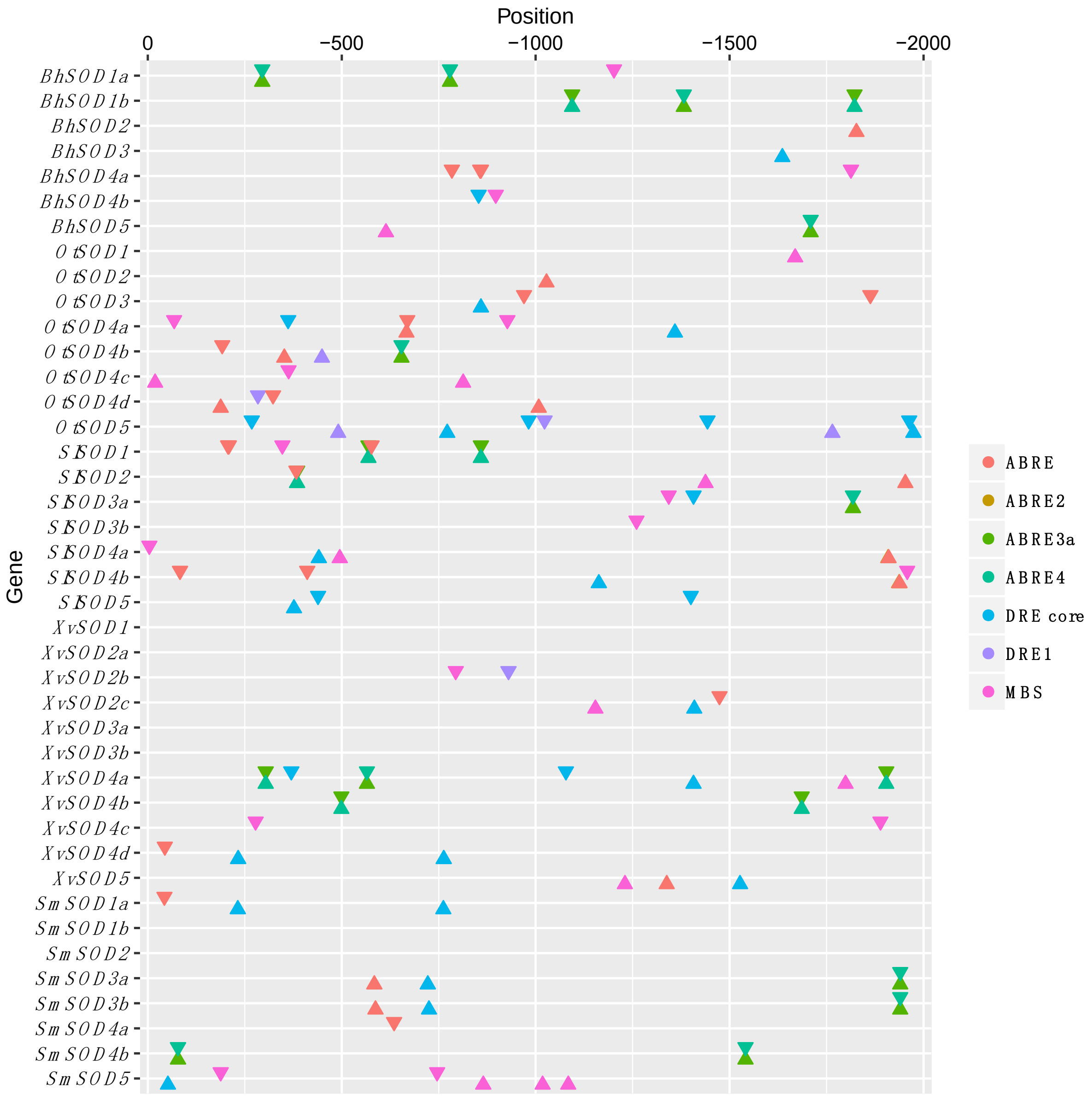

Figure S7. Distribution of drought responsive *cis*-elements in promoters of *SOD* genes. Numbers on the x-axis denote distance from the transcription start site. Colors indicate the different types of *cis*-elements. Downward and upward wedges indicate that the *cis*-element is on the positive or negative strand of the promoter, respectively.
